# Development, Reliability, and Validity of the Preschool Learning Skills Scale: A Tool for Early Identification of Preschoolers at Risk of Learning Disorder in Mainland China

**DOI:** 10.3389/fneur.2022.918163

**Published:** 2022-07-13

**Authors:** Mengmeng Yao, Jing Wang, Panting Liu, Yanru Guo, Yachun Xie, Lei Zhang, Nan Su, Yanwei Li, Dongchuan Yu, Qin Hong, Xia Chi

**Affiliations:** ^1^School of Public Health, Nanjing Medical University, Nanjing, China; ^2^Nanjing Maternity and Child Health Care Hospital, Women's Hospital of Nanjing Medical University, Nanjing, China; ^3^School of Pediatrics, Nanjing Medical University, Nanjing, China; ^4^School of Early-Childhood Education, NanJing XiaoZhuang University, Nanjing, China; ^5^Research Center of Learning Science, Southeast University, Nanjing, China

**Keywords:** learning disorder, preschoolers, early identification, scale development, validity and reliability

## Abstract

**Background:**

Early identification of children at risk of learning disorders (LD) may mitigate the adverse effects of delayed intervention by guiding children to receive preventive services at an earlier age. However, there is no assessment tool for the early identification of children at risk of LD in Mainland China. Therefore, this study aimed to create a Chinese version of the Preschool Learning Skills Scale and investigate its validity and reliability.

**Methods:**

Firstly, a pilot scale was designed based on literature review and expert review. Secondly, a pre-survey of the pilot scale was conducted. In phase 3, a formal survey was carried out to test the reliability and validity of the scale by involving 2,677 preschool children from 7 kindergartens. Data were collected using a checklist for demographic characteristics, the preschool learning skills scale, the Behavior Rating Inventory of Executive Function-Preschool Version (BRIEF-P), and Conners' Rating Scales.

**Results:**

The final scale included 38 items under seven factors. The reliability and validity tests confirmed that the Cronbach's alpha, split-half reliability, and test–retest reliability coefficients of the scale were 0.946, 0.888, and 0.941, respectively. The Spearman correlations of factor-total score ranged from 0.685 to 0.876. The results of criterion-related validity showed a direct and significant association between the preschool learning skills scale with the BRIEF-P (*r* = 0.641, *P* < 0.001) and the cognitive problems factor of Conners' Rating Scales (*r* = 0.564, *P* < 0.001). The model had a good fit (χ^^2^^*/df* = 3.489, RMSEA = 0.047, RMR = 0.024, CFI = 0.912, TLI = 0.900, and IFI = 0.912). Multigroup confirmatory factor analysis supported the structural and measurement invariance on the preschool learning skills scale across gender and grade.

**Conclusions:**

The developed preschool learning skills scale has good reliability and validity, indicating that the scale can be used to identify preschool children at risk of LD and can be recommended for use in clinical research and practice.

## Introduction

Specific learning disorder (LD) is a complex neurodevelopmental disorder (1). According to DSM-5, SLD is a general term that refers to a group of disorders, which may involve persistent difficulties in reading (dyslexia), written expression (dysgraphia), and/or mathematics (dyscalculia), albeit not accounted for by low intelligence (IQ), sensory acuity (e.g., visual problems), poor learning opportunities, or developmental delay (e.g., intellectual disability) (2). DSM-5 describes LD as a neurodevelopmental disorder with a biological origin, including the interaction of genetic, epigenetic, and environmental factors (3, 4). LD is a lifelong disease that may have adverse consequences for children and adults at the educational, social, financial, and professional levels (5).

Studies have shown that the prevalence of LD in the general population ranges from 3 to 12% depending on factors such as different assessment tools, boundaries of measurement, cultural background, gender, age, etc. (6–8). The average age at which children are diagnosed with LD is 9 years (grades 3–4 of primary school). This is when the academic demands rise and exceed the individual's limited capacities, and children begin to appear to face academic difficulties in school (9). However, it is generally believed that LD occurs prior to kindergarten and continues into adulthood (10, 11). Delayed intervention may have adverse and lasting consequences on the acquisition of academic skills. On the contrary, early identification of children at risk of LD may mitigate the adverse effects of delayed intervention by guiding children to receive preventive services at an earlier age (9, 12).

Researchers have developed many behavior checklists for the screening of LD, which are primarily suitable for school-age children, mainly focusing on academic skills such as reading, writing, and arithmetic (13, 14). In addition, there are behavioral checklists for specific disorders, such as Dyslexia Screening Instrument, designed to identify children who exhibit behaviors related to spelling, reading, writing, or language-processing difficulties (15, 16). These screening tools are relatively brief and cost-effective measures to justify a more detailed assessment or diagnostic test (17).

However, preschool age is a crucial period for early identification and intervention of LD. In kindergarten, children with mathematics disorders already have deficits in comparing non-symbolic and symbolic Arabic numbers (18). Several early or pioneer literacy skills measured in preschool-age have demonstrated strong relationships with future decoding and reading comprehension achievement, such as phonological awareness, rapid naming, and oral language (19, 20). Meanwhile, effective, early reading instructions can improve reading outcomes of children with LD (21, 22). Therefore, more and more researchers pay attention to the early recognition of LD in preschool children (23). Some checklists have been developed for early screening of LD in Hong Kong and Taiwan, such as the Hong Kong Learning Behavior checklist for Preschool Children (Parent Version) (24). However, there is no specific assessment tool in mainland China for the early identification of Preschoolers at risk of LD.

Thus, this study aimed to construct a brief, easy-to-use scale to specify the characteristics of LD for parents from mainland China to identify preschoolers at risk of LD at an early stage and to test the validity and reliability of the scale.

## Methods

The Preschool Learning Skills Scale (Supplementary File 1) aimed to develop a brief screening measure for the early identification of preschoolers at a risk for LD. The study was ethically approved by the Institutional Review Committee of Nanjing Medical University.

### Development of the Preschool Learning Skills Scale

The development of the preschool learning skills scale for parents of preschoolers in mainland China followed many stages (25).

In the first stage, based on an extensive review of relevant literature and published questionnaires, as well as on consultations with parents of children with LD, and specialists and teachers who specialize in LD, the primary cognitive and behavioral manifestations of children with LD in the preschool years were determined to identify key components related to LD risk. Finally, a total of 7 dimensions with 71 items were generated from the item pool, which forms the index system framework of the scale, classified into seven categories: attention, memorization, visual perception, auditory perception, motor coordination, verbal competence, and mathematical concept.

In phase 2, a panel of experts in developmental–behavioral pediatrics, psychology, pedagogy, public health, and clinical evaluation (*n* = 5, including the first and corresponding authors) reviewed the bank of items to classify those contents into each key theme to identify gaps and overlaps between items and to figure out the suitability of items for parents of preschool children. The scale was adapted and revised with inappropriate items removed. Finally, 55 items were selected to form the first draft of the preschool learning skills scale. All items were rated on a 5-point Likert scale ranging from never to always.

In phase 3, to ensure the scale's content validity, the questionnaire was reviewed and refined again by a panel of experts (*n* = 8) to determine whether the items comprehensively reflected the key behaviors that could develop into LD in preschool-aged children to establish content validity. Then, the questionnaire was put to an experimental test to render certain that the parents of preschoolers (*n* = 20) understood the items as intended. Furthermore, based on the experimental testing results, the questionnaire was refined by modifying the items which were difficult to understand, had semantic ambiguity, and were prone to ambiguity due to the experimental testing results.

### Pre-Survey of the Preschool Learning Skills Scale

A convenience sample of participants selected 657 preschool children from two kindergartens in Nanjing in December 2020. Exclusionary criteria were children who were diagnosed with a neurodevelopmental disorder such as autism, intellectual disability, or other disabilities. The parents of eligible participants electronically signed the consent document and completed the 55-item learning skills scale (Draft 1 of the preschool learning skills scale) and a demographic survey.

Through the pre-survey, 55 items were scientifically refined in the following methods: critical ratio method, frequency distribution analysis, variation coefficient (CV) method, correlation analysis, Cronbach's alpha coefficient method, and exploratory factor analysis (25). Considering the above six methods comprehensively, if two or more methods excluded an item, the item would be deleted from the scale. Finally, the preschool learning skills scale with 38 items in 7 factors is compiled.

### Reliability and Validity of the Preschool Learning Skills Scale

A total of 2,677 preschool children were selected from 7 kindergartens in Nanjing. Exclusion and inclusion criteria were the same as those for the pre-test of the study. The parents electronically signed the consent document and completed the questionnaire survey within 3 days of receipt of the survey (first assessment T1). In addition to the 38-item learning skills scale (Draft 2 of the preschool learning skills scale) and a demographic survey, 600 parents were randomly selected to extra complete the Behavior Rating Inventory of Executive Function-Preschool Version (BRIEF-P) and Conners' Rating Scales (Conners 3-P) for assessment of criterion-related validity.

The reliability of the scale was assessed using Cronbach's alpha, split-half correlation, and test–retest. A random sample of the parents filled in the preschool learning skills scale 2 weeks after the first survey (T2) to assess test–retest reliability.

For the exploratory and confirmatory factor analysis, data from four kindergartens (*n* = 1,540) were used for the exploratory factor analysis (EFA), and data from three kindergartens (*n* = 1,137) for the confirmatory factor analysis (CFA). The EFA was performed by IBM SPSS Statistics 26.0. The suitability of the data for factorization was evaluated by the value for the Kaiser–Meyer–Olkin (KMO) measure of sampling adequacy and Bartlett's test of sphericity (preferably significant) (26). The EFA was done *via* iterative Maximum Likelihood with Promax Rotation to extract the factors due to the correlation of the factors (27). The criterion for loading and cross-loading was set at 0.4. The CFA was performed with IBM SPSS Amos 22.0. The goodness of model fit was evaluated using the following fit indices: the chi-square goodness of fit (χ^^2^^*/df* ) values, with values <5.0 deemed acceptable; the root-mean-square error of approximation (RMSEA); the root-mean-square residual (RMR) <0.05; the comparative fit index (CFI); the Tucker-Lewis index (TLI); and the incremental fit index (IFI) >0.9 (28, 29). The construct validity of the preschool learning skills scale was examined with the standardized regression coefficients and construct reliability (CR) for convergent validity and the correlation coefficient, and the square root of AVE for discriminant validity.

### Multigroup Confirmatory Factor Analysis of the Preschool Learning Skills Scale

We performed a multigroup confirmatory factor analysis across gender and grade. Subsequently, using the sample from three kindergartens (*n* = 1,137), we conducted a multigroup confirmatory factor analysis to examine the measurement invariance of the seven-factor structure across gender: boys vs. girls. Measurement invariance was also tested across age groups: Group 1 (Junior Class of kindergarten), Group 2 (Middle Class of kindergarten), and Group 3 (Senior Class of kindergarten). The measurement invariance was evaluated using the following fit indices: the change in chi-square values (Δχ2) and the fit indices (RMR, RMSEA, CFI, ΔRMSEA, and ΔCFI) (30, 31). Measurement invariance is supported when ΔRMSEA is <0.015 and ΔCFI is <0.02 (32).

## Results

### Participants

As shown in Table 1, the study included 2,677 preschool children: 1,445 boys and 1,232 girls with a mean age of 5.2 years (SD = 0.9, range 3.5–6.8). There was no significant mean age difference between the genders. Most of the questionnaire fillers were mothers (*n* = 2,090, 78.07%).

**Table 1:** Characteristics of participants (*n* = 2,677).

**Participants**	**Frequency (*n*)**	**Percentage (%)**
**Sex**		
Boys	1,445	53.98
Girls	1,232	46.02
**Age, y**		
3.0–3.9	170	6.35
4.0–4.9	947	35.38
5.0–5.9	861	32.16
6.0–6.9	699	26.11
**Questionnaire fillers**		
Mother	2,090	78.07
Fathers	587	21.93
**Father education**		
Less than high school	129	4.82
High school graduate	350	13.07
Associates degree	614	22.94
Bachelor's degree	1,206	45.05
Master's degree or above	378	14.12
**Mother education**		
Less than high school	157	5.87
High school graduate	354	13.22
Associates degree	605	22.60
Bachelor's degree	1,277	47.70
Master's degree or above	284	10.61

### The Exploratory Factor Analysis of the Preschool Learning Skills Scale

The appropriateness of factor analysis was measured by The Kaiser–Meyer–Olkin (KMO) measure of the sampling adequacy and the Bartlett test of sphericity. The results showed KMO = 0.966 and Bartlett significance *P* < 0.001, indicating that exploratory factor analysis was appropriate.

Seven factors were extracted from the preschool learning skills scale by the EFA (see Table 2). The factor loadings varied from 0.401 to 0.787. The seven factors explained 55.30% of the variance.

**Table 2:** Factor loadings from the exploratory factor analysis of the preschool learning skills scale (*n* = 1,540).

**Item**	**Factors**						
	**A**	**B**	**C**	**D**	**E**	**F**	**G**
**Factor A: Attention**							
Item 1	0.670						
Item 2	0.582						
Item 10	0.694						
Item 11	0.713						
Item 19	0.650						
Item 24	0.593						
Item 35	0.664						
**Factor B: Memorization**							
Item 3		0.458					
Item 6		0.672					
Item 7		0.657					
Item 25		0.401					
**Factor C: Visual perception**							
Item 20			0.432				
Item 22			0.537				
Item 27			0.624				
Item 29			0.696				
Item 34			0.444				
**Factor D: Auditory perception**							
Item 15				0.517			
Item 18				0.579			
Item 28				0.692			
Item 36				0.593			
**Factor E: Motor coordination**							
Item 8					0.583		
Item 12					0.616		
Item 17					0.583		
Item 23					0.432		
**Factor F: Verbal competence**							
Item 4						0.556	
Item 5						0.701	
Item 9						0.598	
Item 13						0.787	
Item 14						0.680	
Item 16						0.605	
Item 21						0.485	
Item 26						0.617	
Item 33						0.701	
Item 37						0.599	
**Factor G: Mathematical concept**							
Item 30							0.534
Item 31							0.650
Item 38							0.673

### Reliability and Validity of the Preschool Learning Skills Scale

The reliability results of PLSS are shown in Table 3 below. Cronbach's alpha coefficient of the preschool learning skills scale was 0.946 and Cronbach's alpha coefficients of the factors ranged from 0.674 to 0.871, all >0.70 except for mathematical concepts, which meant that the scale had good internal consistency reliability. The split-half reliability coefficient was 0.888, and the split-half reliability coefficient of the factors varied between 0.638 and 0.834, indicating that the scale had good internal reliability. The test–retest reliability coefficient was 0.941, and the test–retest reliability coefficient of the factors ranged from 0.774 to 0.939, which reflected excellent test–retest reliability of the scale.

**Table 3:** The reliability of the preschool learning skills scale (*n* = 1,540).

**Factors**	**Cronbach's alpha**	**Split-half**	**Test-retest**
A: Attention	0.835	0.789	0.927
B: Memorization	0.737	0.752	0.856
C: Visual perception	0.716	0.697	0.774
D: Auditory perception	0.757	0.723	0.856
E: Motor coordination	0.703	0.713	0.917
F: Verbal competence	0.871	0.834	0.939
G: Mathematical concept	0.674	0.638	0.842
Total score	0.946	0.888	0.941

The Spearman correlations of the factor-total score ranged from 0.685 to 0.876. The Spearman correlations of the interfactor varied between 0.417 and 0.716 (Table 4); the correlation coefficients among factors A, B, C, D, and F were beyond 0.6, but <0.8, indicating that the correlation between factors had reached an acceptable level. Moreover, all Spearman correlations of the factor-total score were more significant than the interfactor. The results of the correlation analysis showed a direct and significant association of the preschool learning skills scale with the Behavior Rating Inventory of Executive Function-Preschool Version (*r* = 0.641, *P* < 0.001) and the cognitive problems factor of Conners' Rating Scales (*r* = 0.564, *P* < 0.001).

**Table 4:** Spearman correlations of interfactor and factor-total score (*n* = 1,540).

**Factors**	**Factors**						
	**A**	**B**	**C**	**D**	**E**	**F**	**G**
A: Attention	1						
B: Memorization	0.651^***^	1					
C: Visual perception	0.553^***^	0.577^***^	1				
D: Auditory perception	0.571^***^	0.614^***^	0.620^***^	1			
E: Motor coordination	0.512^***^	0.502^***^	0.499^***^	0.567^***^	1		
F: Verbal competence	0.563^***^	0.668^***^	0.665^***^	0.716^***^	0.569^***^	1	
G: Mathematical concept	0.417^***^	0.490^***^	0.569^***^	0.545^***^	0.488^***^	0.573^***^	1
Total score	0.803^***^	0.804^***^	0.787^***^	0.817^***^	0.712^***^	0.876^***^	0.685^***^

*** *P < 0.001*.

### The Confirmatory Factor Analysis of the Preschool Learning Skills Scale

**Figure 1:**
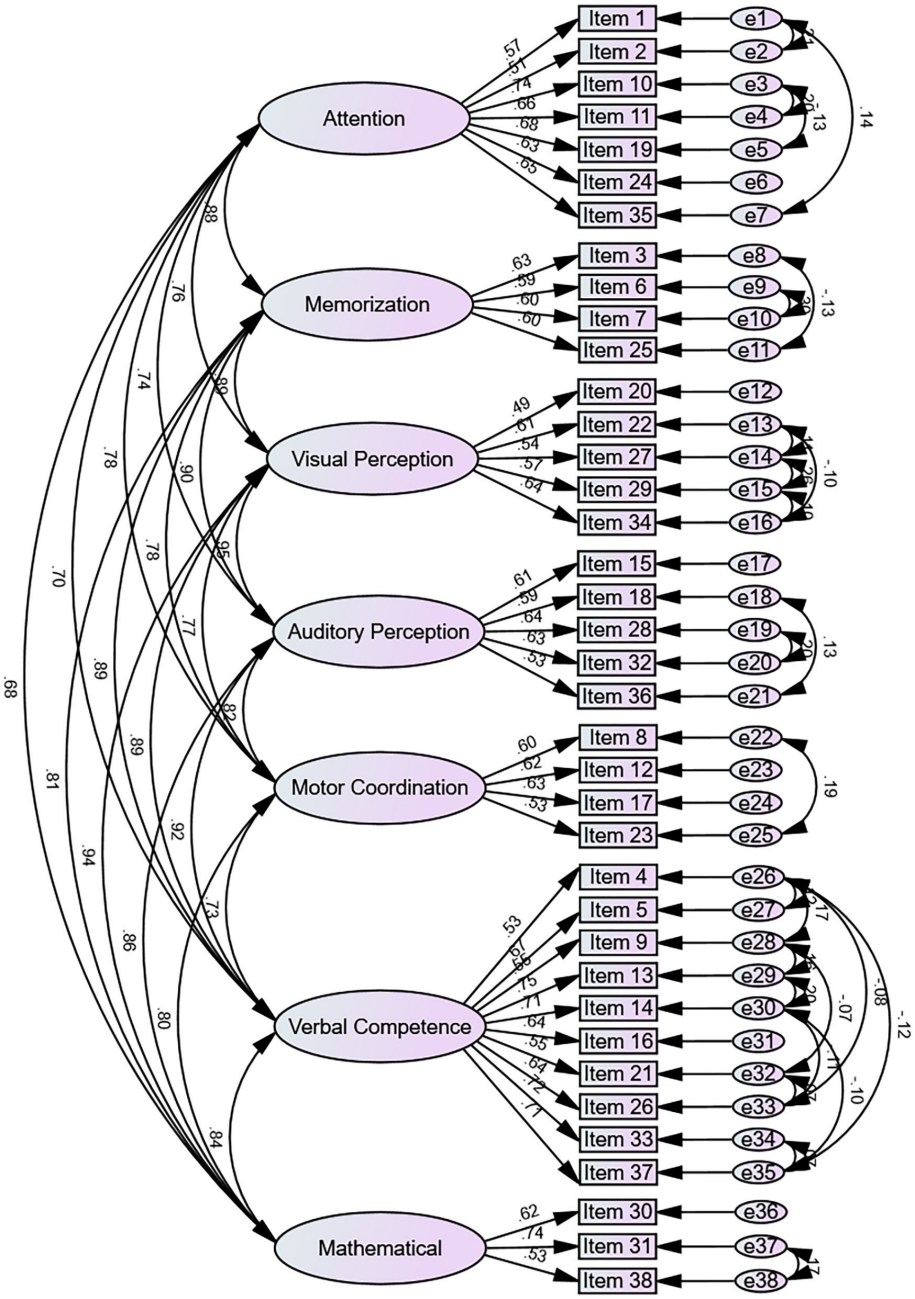
The standardized path coefficients of the preschool learning skills scale (*n* = 1,137).

Figure 1 shows the factor structure and model fit of the preschool learning skills scale using CFA. The CFA of the seven-factor model of the preschool learning skills scale showed that the χ^^2^^*/df* = 3.489, the RMSEA was 0.047, the RMR was 0.024, the CFI was 0.912, the TLI was 0.900, and the IFI was 0.912. These results indicated that the goodness-of-fit index of the model was valid. The standardized regression coefficients of each item of the preschool learning skills scale ranged from 0.490 to 0.747. These values were more than 0.4 (Table 5) and CR values also were more than 1.965 (*P* < 0.001), which indicated that the items corresponding to each latent variable were highly representative. The discriminant validity of the preschool learning skills scale was evaluated using correlation coefficients among seven factors (Table 6). Correlations ranged from 0.129 to 0.211 among seven factors, all of which were significantly correlated (*P* < 0.001). The correlation coefficients were all less than the square root of the corresponding AVE. These results were satisfactory and indicated adequate discriminant validity in the study.

**Table 5:** Standardized regression coefficients of the preschool learning skills scale (*n* = 1,137).

**Item**		**Factor**	**Unstandardized estimate**	**S.E**.	**C.R**.	**Standardized estimate**
Item 1	<–	A	1.000			0.571
Item 2	<–	A	0.902	0.057	15.751	0.510
Item 10	<–	A	1.203	0.068	17.621	0.739
Item 11	<–	A	1.259	0.076	16.556	0.660
Item 19	<–	A	1.039	0.062	16.864	0.679
Item 24	<–	A	1.028	0.063	16.242	0.629
Item 35	<–	A	1.047	0.058	17.999	0.650
Item 3	<–	B	1.000			0.626
Item 6	<–	B	0.988	0.058	16.964	0.589
Item 7	<–	B	0.965	0.056	17.169	0.598
Item 25	<–	B	0.846	0.052	16.309	0.602
Item 20	<–	C	1.000			0.490
Item 22	<–	C	1.538	0.107	14.318	0.607
Item 27	<–	C	1.187	0.089	13.387	0.539
Item 29	<–	C	1.238	0.089	13.833	0.571
Item 34	<–	C	1.184	0.080	14.758	0.643
Item 15	<–	D	1.000			0.607
Item18	<–	D	0.988	0.059	16.693	0.586
Item 28	<–	D	0.801	0.045	17.958	0.645
Item 32	<–	D	0.854	0.049	17.589	0.628
Item 36	<–	D	0.963	0.063	15.356	0.529
Item 8	<–	E	1.000			0.600
Item 12	<–	E	0.765	0.048	15.798	0.620
Item 17	<–	E	0.925	0.058	15.890	0.625
Item 23	<–	E	0.717	0.046	15.715	0.529
Item 4	<–	F	1.000			0.534
Item 5	<–	F	1.240	0.071	17.516	0.672
Item 9	<–	F	1.094	0.069	15.805	0.548
Item 13	<–	F	1.221	0.070	17.384	0.747
Item 14	<–	F	1.032	0.061	16.909	0.714
Item 16	<–	F	1.072	0.067	15.922	0.636
Item 21	<–	F	0.970	0.067	14.455	0.549
Item 26	<–	F	1.132	0.073	15.449	0.645
Item 33	<–	F	1.184	0.069	17.095	0.723
Item 37	<–	F	1.257	0.078	16.105	0.708
Item 30	<–	G	1.000			0.624
Item 31	<–	G	1.168	0.061	19.069	0.736
Item 38	<–	G	1.171	0.079	14.800	0.535

**Table 6:** Discriminant validity of the preschool learning skills scale (*n* = 1,137).

	**A**	**B**	**C**	**D**	**E**	**F**	**G**
A: Attention	(-)						
B: Memorization	0.211^***^	(-)					
C: Visual perception	0.131^***^	0.145^***^	(-)				
D: Auditory perception	0.162^***^	0.186^***^	0.142^***^	(-)			
E: Motor coordination	0.209^***^	0.200^***^	0.142^***^	0.191^***^	(-)		
F: Verbal competence	0.148^***^	0.179^***^	0.129^***^	0.169^***^	0.164^***^	(-)	
G: Mathematical concept	0.156^***^	0.178^***^	0.148^***^	0.173^***^	0.198^***^	0.163^***^	(-)
Square root of AVE	0.638	0.604	0.573	0.600	0.595	0.652	0.637

*** *P < 0.001*.

### Measurement Invariance Across Gender and Grade

To examine the measurement invariance of the preschool learning skills scale across both gender and grade, we used a multigroup confirmatory factor analysis approach, which assesses the measurement invariance across two or more groups by using a series of increasingly stringent, nested models.

Table 7 presented that the fit indices for Model 1 (configural invariance) indicated that the seven-factor measurement model of the preschool learning skills scale had an acceptable fit within each gender group (RMR = 0.027; RMSEA = 0.036; CFI = 0.899). Based on the indices of practical fit and the change in RMSEA and CFI criterion recommended, our results concluded that Model 2 (Metric invariance), Model 3 (Scalar invariance), and Model 4 (Strict factorial) fit nearly as well as Model 1 (RMR <0.05; RMSEA <0.05; CFI >0.8; ΔRMSEA <0.015; and ΔCFI <0.02), supporting measurement invariance across gender groups.

**Table 7:** Multigroup CFA Fit indices for the preschool learning skills scale across gender (*n* = 1,137).

**Model**	**χ2**	***df***	**Δχ2**	**Δ*df***	***P***	**RMR**	**RMSEA**	**CFI**	**ΔRMSEA**	**ΔCFI**
Model 1 (configural invariance)	3,011.386	1,238.000				0.027	0.036	0.899		
Model 2 (metric invariance)	3,062.051	1,269.000	50.665	31.000	0.014	0.029	0.035	0.898	−0.001	−0.001
Model 3 (scalar invariance)	3,107.914	1,297.000	96.528	59.000	0.001	0.040	0.035	0.897	−0.001	−0.002
Model 4 (strict factorial)	3,251.864	1,360.000	240.478	122.000	0.000	0.041	0.035	0.892	−0.001	−0.007

Subsequently, measurement invariance across grades was examined. As Table 8 showed, the configural invariance was supported by the acceptable absolute fit indices (RMR = 0.029; RMSEA = 0.031; CFI = 0.887). In addition, also measurement invariance (at the metric, scalar, and strict factorial level) across grades was present because all absolute and relative fit indices were acceptable. All these results indicated a reasonable level of structural invariance and measurement invariance in all three grade groups of the preschool learning skills scale.

**Table 8:** Multigroup CFA fit indices for the preschool learning skills scale across grade (*n* = 1,137).

**Model**	**χ2**	***df***	**Δχ2**	**Δ*df***	***P***	**RMR**	**RMSEA**	**CFI**	**ΔRMSEA**	**ΔCFI**
Model 1 (configural invariance)	3,830.508	1,857.000				0.029	0.031	0.887		
Model 2 (metric invariance)	3,884.913	1,888.000	54.405	31.000	0.006	0.032	0.031	0.886	0.000	−0.001
Model 3 (scalar invariance)	3,939.602	1,916.000	109.094	59.000	0.000	0.036	0.031	0.885	0.000	−0.002
Model 4 (strict factorial)	4,176.705	1,979.000	346.197	122.000	0.000	0.037	0.031	0.875	0.000	−0.012

## Discussion

Learning disorders refers to a group of disorders characterized by significant difficulties in listening, reading, speaking, writing, attention, memorization, and coordination. These difficulties range from mild to severe. Researchers have made consistent efforts to identify and intervene early to ensure that children receive assistance prior to having poor learning experiences and prevent other problems that may affect their learning abilities (16, 18, 21). As to early identification, it is believed that parents are the ones who closely observe children in parent–child interactions to identify behavioral indicators of LD, such as literacy problems and specific cognitive deficits. Thus, we developed the preschool learning skills scale as a parental checklist to provide information on the characteristics of children with LD at preschool age and as a screening measure to make a more detailed follow-up assessment of children at risk of LD.

Different manifestations of LD can be seen at various ages and as a result of varying learning demands. Delays in speech and language development, numerical and symbolic concepts, motor coordination, and auditory and visual perception are early indicators of children who may have LD (33–35). These indicators may occur concomitantly with attention, memorization, or self-regulation problems. The Hong Kong Learning Behavior checklist for Preschool Children (Parent Version), which was developed by Hong Kong Specific Learning Difficulties Research Team in 2006, identified preschoolers at the risk of learning difficulties in seven aspects: language ability, learning ability, writing performance, attention, memorization, sequencing ability, spatial awareness, and motor coordination (24). However, the scale was specifically validated for the population of Hong Kong, and mainly aimed at the early identification of dyslexia and dysgraphia. The spoken language of Hong Kong includes Cantonese and English, and the scale contains some items to check English ability. Besides, the education system of Hong Kong is different from that of mainland China. The teaching of literacy and handwriting begins in early childhood, much earlier than in the Mainland, so there are many items about the ability to learn Chinese (such as reading or interest in words) and writing performance in that scale. Therefore, the scale is not wholly applicable to the early recognition of LD for Mainland children, but the theoretical framework and some items are still worth our reference.

In this study, we first defined the connotation and characteristics of LD by reviewing the related researches. A literature search was performed using the following terms: “learning ability,” “LD,” “dyslexia,” “mathematical disorder,” “kindergarten,” and “preschool children” in PubMed, EMBASE, Web of Science, and MEDLINE, CNKI, and Wanfang databases. In the retrieved literature, the behavioral manifestations and characteristics of children with LD were perused, as well as related guidelines, expert consensus, systematic evaluation, and original research on screening and diagnosis of LD. Then, we summarized and compared the scales or tools for early identification of LD in domestic and foreign studies, such as the Pupil Rating Scale Revised (PRS) questionnaire, Hong Kong Learning Behavior checklist for Preschool Children (Parent Version), East Asia-Pacific Early Child Development Scales, South African Early Learning Outcomes Measure, etc., to clear and definite the methods, structures, and indicators of early identification of LD. Finally, we studied the localization theory of early identification for LD in Mainland China. Through in-depth interviews with experts in child psychology, developmental-behavioral pediatrics, teachers, and parents of children with LD, their attitudes, views, and experiences toward the early identification of LD in the preschool age were summarized. From these, the preschool learning skills scale, which is the first scale for early identification of preschoolers at risk of LD in Mainland China, establishes the item pool for the initial experimental checklist based on the theoretical framework of the following: attention, memorization, visual perception, auditory perception, motor coordination, verbal competence, and mathematical concept.

Our research conducted psychometric evaluation through the critical ratio method, frequency distribution analysis, variation coefficient (CV) method, correlation analysis, Cronbach's alpha coefficient method, and exploratory factor analysis, suggesting the retention of 38 items to be included in the revised checklist. To evaluate the reliability and validity of the 38-item preschool learning skills scale, we conducted a questionnaire survey among 2,677 preschool children from 7 kindergartens. Overall, our results demonstrate that the developed preschool learning skills scale had good reliability and validity, and showed an excellent fit of the seven derived factors *via* exploratory and confirmatory factor analysis. After establishing a robust factor structure within a sample, it is essential to know whether this factor structure is also applicable across samples or groups, as this “invariance” is the only condition that can be allowed to investigate group differences, for instance, between different genders and ages, and so on (36). Given the difference between the scale scores of boys and girls (*P* < 0.001) and three grades of kindergarten (*P* < 0.001), it is crucial to establish measurement invariance across gender and grade to elucidate if the preschool learning skills scale's sensitivity can identify preschool children at risk of LD. The results of the multigroup confirmatory factor analysis indicated a reasonable level of structural invariance and measurement invariance across gender and grade of the preschool learning skills scale. Therefore, as a screening scale, the preschool learning skills scale was considered to provide information on the cognitive weaknesses of LD and could be used to screen for children at risk of LD for further assessment or preventive interventions.

Nevertheless, further research is needed. Since this study relied on parent reports, it will be necessary for future research to explore the correspondence between parental reports with other reports (such as teachers) and observations of clinicians or educational psychologists (37, 38). More specifically, it will be necessary to further establish the validity of the preschool learning skills scale in the prediction of LD by using multimodal methods and exploring the relationship between the preschool learning skills scale with experimental and behavioral paradigms, such as rapid automatized naming, phonological awareness, letter knowledge, and short-term verbal memorization (19, 39).

Despite these limitations, the preschool learning skills scale has the potential to be a reliable measure that provides a scientific basis for early identification and intervention of LD in preschool age.

## Conclusions

To our knowledge, this is the first study to develop a brief, easy-to-use questionnaire to describe the characteristics of LD in preschool-age for early identification of children at risk of LD. Overall, as an instrument, the developed preschool learning skills scale has good reliability and validity, which indicates that the scale can be used for the early identification of preschool children at risk of LD and can be recommended for use in clinical research and practice. However, study findings are limited to the early identification of preschool children at the risk of LD, and the evaluation of the predictability of the scale is needed. Further research is needed to evaluate the validity of the scale by examining the relationship between the preschool learning skills scale and objective predictors of LD, such as rapid automatized naming, phonological awareness, letter knowledge, and short-term verbal memorization.

## Data Availability Statement

The raw data supporting the conclusions of this article will be made available by the authors, without undue reservation.

## Ethics Statement

The studies involving human participants were reviewed and approved by the Medical Ethics Committee in the Nanjing Maternity and Child Health Care Hospital. Written informed consent to participate in this study was provided by the participants' legal guardian/next of kin.

## Author Contributions

MY: conceptualization, methodology, and writing—original draft preparation. JW: data curation, formal analysis, and writing—original draft preparation. PL: methodology and data curation. YX: validation. YG: data collection. LZ: data collection and validation. NS: language proofreading. YL: funding acquisition. DY: methodology. QH: supervision and writing—reviewing and editing. XC: funding acquisition and writing—reviewing and editing. All authors reviewed the manuscript. All authors contributed to the article and approved the submitted version.

## Funding

This work was supported by the High Level Talents of Jiangsu Province (Project No. WSN-165/WSW-125), the Key Medical Science and Technology Development Projects in Nanjing (Project No. ZKX 18044), the Medical Science and Technology Development Projects in Nanjing (Project No. YKK 19115), Specialized Disease Cohort Study of Nanjing Medical University (Project No. NMUC2018014A), the Key Young Medical Talents of Jiangsu Province (Project No. QNRC2016100), and the National Natural Science Foundation of China (Project No. 32000759).

## Conflict of Interest

The authors declare that the research was conducted in the absence of any commercial or financial relationships that could be construed as a potential conflict of interest.

## Publisher's Note

All claims expressed in this article are solely those of the authors and do not necessarily represent those of their affiliated organizations, or those of the publisher, the editors and the reviewers. Any product that may be evaluated in this article, or claim that may be made by its manufacturer, is not guaranteed or endorsed by the publisher.

## Abbreviations

LD: learning disorder
BRIEF: behavior rating inventory of executive function
EFA: exploratory factor analysis
CFA: confirmatory factor analysis
*χ^2^/df*: chi-square goodness of fit values
RMSEA: root-mean-square error of approximation
RMR: root-mean-square residual
CFI: comparative fit index
TLI: tucker-lewis index
IFI: incremental fit index
CR: composite reliability
AVE: average variance extracted.
