# Supplementary material for: Development, Reliability, and Validity of the Preschool Learning Skills Scale: A Tool for Early Identification of Preschoolers at Risk of Learning Disorder in Mainland China
Source: Front Neurol. 2022 Jul 13;13:918163. doi: 10.3389/fneur.2022.918163 (PMC9326052; doi:10.3389/fneur.2022.918163)
Supplement: Supplementary file 1 [file Data_Sheet_1.docx]

**The Preschool Learning Skills Scale (Parent Version)**

Mark the most appropriate item with “√” based on your child’s daily behaviors.

“Never” means 0% of the time.

“Occasionally” means 25% of the time.

“Sometimes” means 50% of the time.

“Often” means 75% of the time.

“Usually” means most of the time.

| Items | Never | Occasionally | Sometimes | Often | Usually |
| --- | --- | --- | --- | --- | --- |
| 1. Can be easily distracted by external disturbance from the task that is supposed to be done, and may have difficulty completing the task, which leaves the impression of “fine start, poor finish.” |  |  |  |  |  |
| 2. Appears to be careless and to lose things easily in daily life |  |  |  |  |  |
| 3. Repeats the same mistakes over and over again even though they have been pointed out |  |  |  |  |  |
| 4. Cannot distinguish subtle pronunciation differences, such as “小姨子” and “小椅子”, “毛笔” and “眉笔”. |  |  |  |  |  |
| 5. Says sentences or words in reverse order and makes grammatical mistakes |  |  |  |  |  |
| 6. Needs more time and energy to learn nursery rhymes or short verses, and forgets them soon after they have been acquired |  |  |  |  |  |
| 7. Cannot retell the information that has just been heard, such as phone number, name, address, etc. |  |  |  |  |  |
| 8. Shows physical clumsiness, such as having difficulty with whole-body movement coordination during ball bouncing and rope skipping |  |  |  |  |  |
| 9. Utters sounds indistinct and may easily confuse the words with similar pronunciations, such as uttering “t” as “d”, “j” as “x”. |  |  |  |  |  |
| 10. Appears to be absent-minded in daily activities, such as during face-to-face communications or group activities in kindergarten |  |  |  |  |  |
| 11. Has difficulty staying focused on the activities which are not in his/her interest |  |  |  |  |  |
| 12. Shows inflexibility in hand-eye coordination, such as being unable to use scissors or chopsticks well or to complete coloring/painting activities |  |  |  |  |  |
| 13. Cannot successfully use words to convey ideas |  |  |  |  |  |
| 14. Cannot paraphrase or summarize the words or stories heard |  |  |  |  |  |
| 15. Seems unable to clearly hear the other during conversations, so the child may ask "Hah?" or "What?" |  |  |  |  |  |
| 16. Cannot accurately say the name of common articles or colors, and usually names them by vague words, such as “this”, “that”, “it” etc. |  |  |  |  |  |
| 17. Has difficulty managing personal life independently, such as acting slowly when putting on/taking off socks or clothes, buttoning up, tying shoelaces, or even being unable to complete these tasks |  |  |  |  |  |
| 18. Cannot precisely hear what the adult says when the adult is talking to the child in an environment where many people are talking |  |  |  |  |  |
| 19. Gets stuck on details and loses sight of the whole picture |  |  |  |  |  |
| 20. Has difficulty figuring out orientations, such as front/back, up/down, left/right, etc. |  |  |  |  |  |
| 21. Cannot recall or describe what has happened with peers in kindergarten |  |  |  |  |  |
| 22. Loses track of what has just been read, and may miss words or skip lines |  |  |  |  |  |
| 23. Has difficulty maintaining balance and may easily fall over |  |  |  |  |  |
| 24. Turns a blind eye and turns a deaf eye to things |  |  |  |  |  |
| 25. Has difficulty recalling what was remembered in the past |  |  |  |  |  |
| 26. May pause, lose words, or change subjects when expressing ideas |  |  |  |  |  |
| 27. Reverses words when reading, such as misreading “21” as “12” |  |  |  |  |  |
| 28. Does not understand the conversations at a normal speed and can only comprehend unless words are slowly repeated, which shows a slow reaction. |  |  |  |  |  |
| 29. May confuse similar-looking numbers or letters, such as seeing 3 as 5, 6 as 9, b as d, d as q, etc. |  |  |  |  |  |
| 30. Has difficulty with puzzles or lego construction play |  |  |  |  |  |
| 31. Faces more challenges when dealing with counting compared with peers ( One-to-One Correspondence, Cardinality, Order Irrelevance) |  |  |  |  |  |
| 32. Can remember simple operational procedures only after they have been repeated many times, such as operating a toy car, etc. |  |  |  |  |  |
| 33. Hard to be understood by strangers mainly because of incomplete sentences, lack of linking words, reversed cause-and-effect link |  |  |  |  |  |
| 34. Cannot find things even with hints, such as clothes, shoes, toys, books, etc. |  |  |  |  |  |
| 35. Can be easily distracted or disturbed by irrelevant stimulations |  |  |  |  |  |
| 36. Must stop what is currently doing to be able to listen to the conversations in the surrounding environment |  |  |  |  |  |
| 37. Has a smaller vocabulary compared with peers and uses simple, limited words with few adjectives or descriptions |  |  |  |  |  |
| 38. Cannot do simple addition or subtraction with tools such as fingers, blocks, etc. |  |  |  |  |  |
